# Objects Classification by Learning-Based Visual Saliency Model and Convolutional Neural Network

**DOI:** 10.1155/2016/7942501

**Published:** 2016-10-10

**Authors:** Na Li, Xinbo Zhao, Yongjia Yang, Xiaochun Zou

**Affiliations:** ^1^School of Computer Science, Northwestern Polytechnical University, Xi'an, China; ^2^School of Electronics and Information, Northwestern Polytechnical University, Xi'an, China

## Abstract

Humans can easily classify different kinds of objects whereas it is quite difficult for computers. As a hot and difficult problem, objects classification has been receiving extensive interests with broad prospects. Inspired by neuroscience, deep learning concept is proposed. Convolutional neural network (CNN) as one of the methods of deep learning can be used to solve classification problem. But most of deep learning methods, including CNN, all ignore the human visual information processing mechanism when a person is classifying objects. Therefore, in this paper, inspiring the completed processing that humans classify different kinds of objects, we bring forth a new classification method which combines visual attention model and CNN. Firstly, we use the visual attention model to simulate the processing of human visual selection mechanism. Secondly, we use CNN to simulate the processing of how humans select features and extract the local features of those selected areas. Finally, not only does our classification method depend on those local features, but also it adds the human semantic features to classify objects. Our classification method has apparently advantages in biology. Experimental results demonstrated that our method made the efficiency of classification improve significantly.

## 1. Introduction

Objects classification is one of the most essential problems in computer vision. It is the basis of many other complex vision problems, such as segmentation, tracking, and action analysis. And objects classification has wide application in many fields, such as security, transportation, and medicine. Thus, computer automatic classification technology can lighten the burden of people and change people's life style.

**Figure 1:**
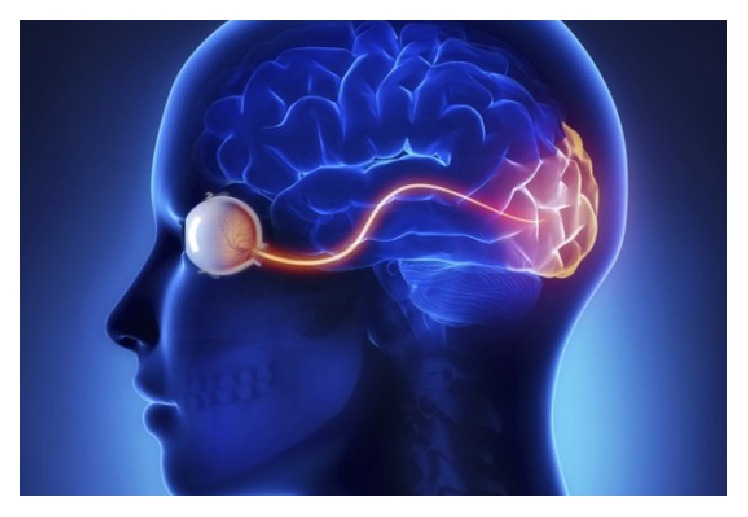
The picturesque processing of humans classifying different objects. Person firstly selects information by visual pathway, and then his nervous system uses this selected information to make correct decision without needing extensive training [6].

**Figure 2:**
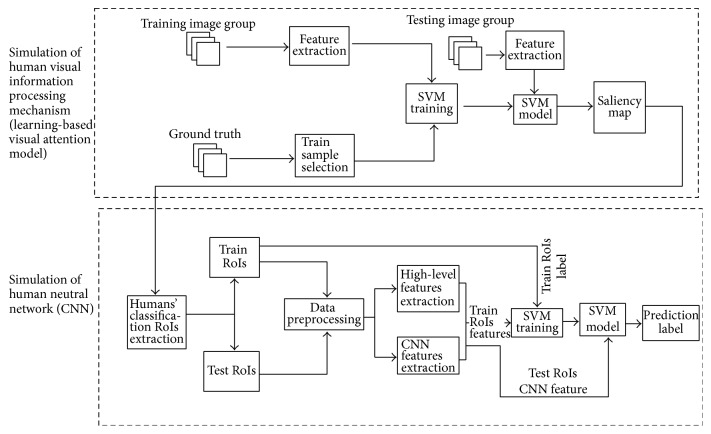
The algorithm flow chart of this paper. We establish a learning-based visual saliency model to simulate human visual information processing mechanism and then obtain saliency map which can be used to get the humans' classification RoIs. CNN is used to simulate human neutral network, and the humans' classification RoIs is CNN's input. After the processing of CNN, we obtain the result of classification which is close to humans.

Humans have the powerful ability of visual perception and objects classification. When they classify different objects, they firstly select information by visual pathway, and then their nervous system make correct decision without needing extensive training by using this selected information (Figure 1). If computer can mimic the ability of humans, computer automatic classification technology will be improved greatly. To achieve this assumption, we combine simulation of human visual information processing mechanism and simulation of human neutral network (Figure 2).

Referring to the research results from cognitive psychology and neuroscience, we can build learning-based visual attention model as human visual information processing mechanism. Most models of attention [1–3] are biologically inspired. But some of them are only based on a bottom-up computational model, which does not match the human behavior. Other models of attention such as Cerf et al. [4] combine low-level visual features and high-level visual features, but most of them were under the “free-viewing”; it cannot be used to analyze and predict the region of interest when people classify different objects. To address this problem, we build a task-based and learning-based visual attention model combining low-level and high-level image features to obtain the humans' classification RoI (region of interest).

Deep learning is good to disentangle abstractions and pick out which features are useful for learning like human brain does, so we can use deep learning method to simulate the human neutral network. Convolutional neural network (CNN) as one of methods of deep learning can be used to solve classification problem. CNN was inspired by biological processes [5], which is a type of feed-forward artificial neural network. It is inspired by the organization of the animal visual cortex, whose individual neurons are arranged in such a way that they respond to overlapping regions tiling the visual field. Compared to other image classification algorithms, CNN uses relatively little preprocessing. The lack of dependence on prior knowledge and human effort in designing features is a major advantage of CNN, which lead CNN to be more suitable for solving computer automatic classification problem.

In this paper, we make five contributions. Firstly, for learning common people visual behaviors when they classify different objects, we established an eye-tracking database and recorded eye-tracking data of 10 viewers on 300 images. Secondly, to simulate human visual information processing mechanism when they were asked to classify different objects, we used EDOC database as training and testing examples to learn a learning-based visual attention model based on low-level and high-level image features and then analyzed and predicted the humans' classification RoIs. Next, seeing that the CNN is inspired by biological processes and has remarkable advantages, we established a CNN framework to simulate the human brain's processing of classification. But, unlike traditional CNN, we use RoIs predicted from our learning-based visual attention model as the input of CNN, and thus it will be more close to human. Furthermore, for improving the biological advantages of our computer automatic classification method, we combine the high-level features also used in our visual attention model with local features gained by our CNN network to classify objects by SVM. Finally, we established big database ImageSix, including 6000 images to testify the robustness of our classification method.

And all experimental results showed that our method made the efficiency of classification improve significantly.

## 2. Related Work

Objects classification is one of hot problems in computer vision. Humans recognize a multitude of objects in images with little effort; however, this task is still a challenge for computer vision systems. Many approaches to the task have been implemented over many decades, such as approaches based on CAD-like object models [7], appearance-based methods [8–11], feature-based methods [12–14], and genetic algorithm [15]. These traditional approaches perform well in some fields, but they are not suitable for multiple-classes objects classification. Currently, the best algorithms for this problem are based on convolutional neural networks. An illustration of their capabilities is given by the ImageNet Large Scale Visual Recognition Challenge; this is a milestone in object classification and detection, with millions of images and hundreds of object classes. And performance of convolutional neural networks on the ImageNet tests is now close to that of humans.

For being more close to humans, it is very significant to bring visual saliency model to CNN as our method, because common CNN ignores the idea that human visual system has a major part to select information before classification. So we develop a learning-based visual attention model.

In the past few years, there were many researches on human eye movements, and many saliency models based on various techniques with compelling performance exist, but most of them were under the “free-viewing.” One of the most influential ones is a pure bottom-up attention model proposed by Itti et al. [16], based on the feature integration theory [17]. In this theory, an image is decomposed into low-level attributes such as color, orientation, and intensity. Based on the idea of decorrelation of neural responses, Garcia-Diaz et al. [18] proposed an effective model of saliency known as Adaptive Whitening Saliency (AWS). Another class of models is based on probabilistic formulation. Zhang et al. [19] put forward SUN (Saliency Using Natural statistics) model in which bottom-up saliency emerges naturally as the self-information of visual features. Similarly, Torralba [20] proposed a Bayesian framework for visual search which is also applicable for saliency detection. Graph Based Visual Saliency (GBVS) [21] is another method based on graphical models. Machine learning approaches have also been used in modeling visual attention by learning models from recorded eye fixations. For learning saliency, Schölkopf et al. [22] used image patches and Tilke et al. [23] used a vector of several features at each pixel.

These computational models have been used to characterize RoIs in natural images, but their use in classification has remained very limited. But their features extraction method has been proven certainly effective. When we built a visual attention model for classification problem, we learnt these computational models' features extraction method for guidance.

## 3. Learning a Saliency Model for Objects Classification

### 3.1. Database of Eye-Tracking Data

**Figure 3:**
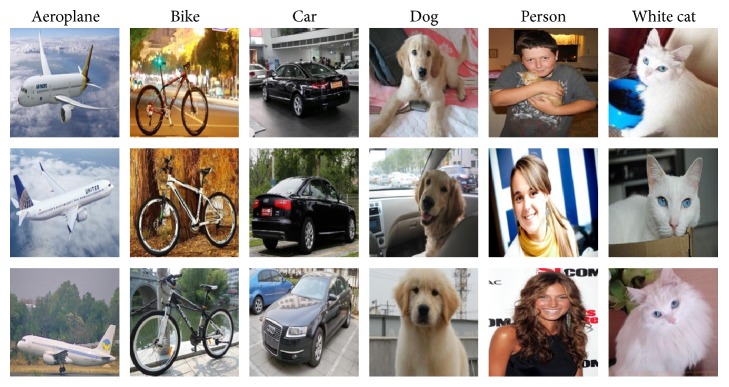
Images. A sample of the 300 images of EDOC. Though they were shown at original resolution and aspect ratio in the experiment, they have been resized for viewing here.

For learning common people visual behaviors when they classify different objects and recording their eye-tracking data, we established an eye-tracking database, including six kinds of objects such as aeroplanes, bikes, cars, dogs, persons, and white cats, called EDOC database (eye-tracking database for objects classification) (Figure 3). The EDOC allows quantitative analysis of fixation points and provides ground truth data for saliency model research as well as labels for each class. Compared with several eye-tracking datasets that are publicly available, the main motivation of our new dataset is for objects classification.

**Figure 4:**
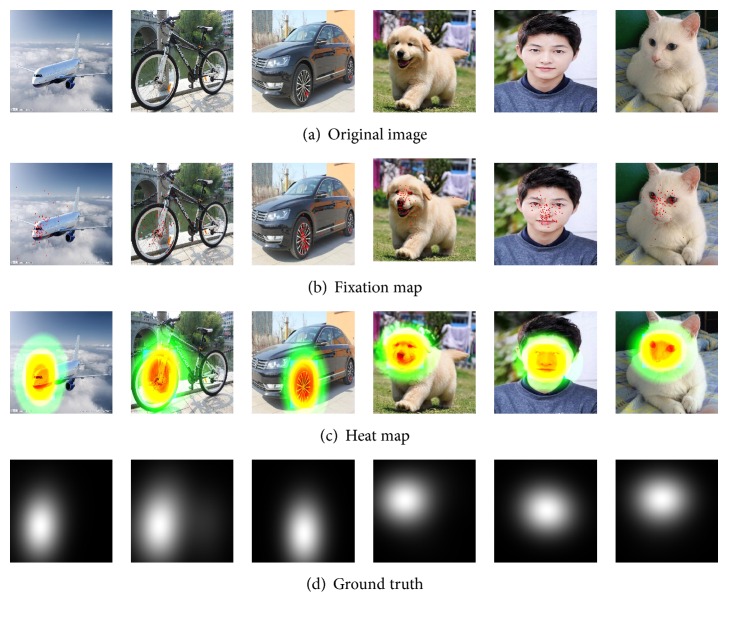
We collected eye-tracking data on 300 images from ten subjects. The first row is the sample of original images (a) in EDOC. Gaze tracking paths and fixation locations are recorded in the second row (b). The third row (c) heat maps show RoIs according to (b). A continuous ground truth (d) is found by convolving Gaussian over the fixation locations of all subjects.

The purpose of the current analysis was to model the classification process of visual selection of relevant regions in different objects images. We collected 50 images for each class of objects and 300 images (Figure 4(a)) altogether, which are stored in JPEG format. And we recorded eye-tracking data from ten subjects, including 5 females and 5 males, whose age range from 12 to 40. Subjects were asked to view these images to find the most representative regions of each class (Figure 4(b)), which can be used to differentiate the six classes objects.

We used a Tobii TX300 Eye Tracker device to record eye movements, which is at a sample rate of unique combination of 300 Hz. The TX300 Eye Tracker device has very high precision and accuracy and robust eye tracking; besides, it also has compensation for large head movements extending the possibilities for unobtrusive research of oculomotor functions and human behavior. Although it has a variety of researcher profiles, subjects can use the system without needing extensive training.

In the experiments, each image was presented for 5 s followed by a rapid and automatic calibration procedure. To ensure high-quality tracking results, we checked camera calibration every 10 images. During the first 1 s viewing, subjects maybe free viewed the images, so we discarded the first 1 s viewing tracking results of each subject. In order to obtain a continuous ground truth of an image from the eye-tracking data of a subject, we convolved a Gaussian filter across the subject's fixation locations, similar to the “landscape map.” We overlapped the eye-tracking data collected from all subjects (Figure 4(c)) and then generated ground truth of the average locations (Figure 4(d)).

### 3.2. Learning-Based Visual Attention Model

**Figure 5:**
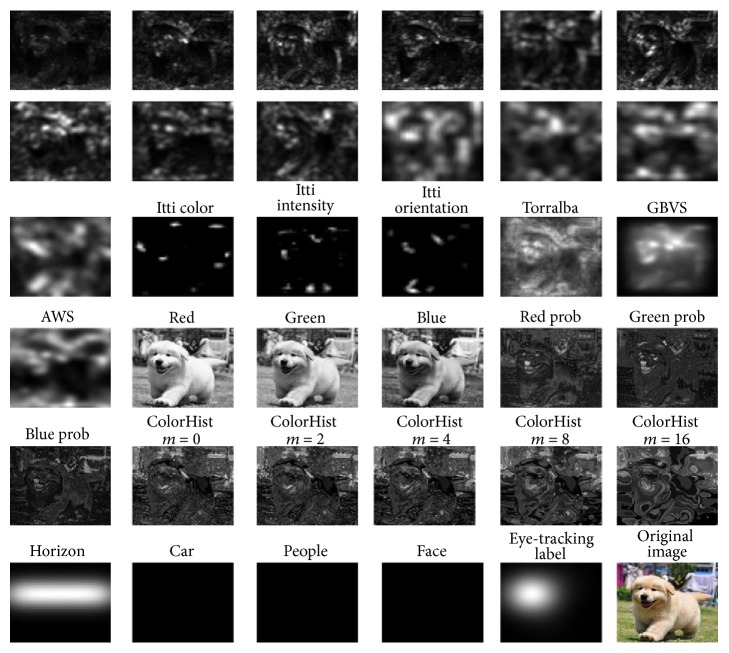
Features. A sample image (bottom right) and 35 of the features that we use to train the model. These include subband features, Itti and Koch saliency channels, three simple saliency models described by Torralba and GBVS and AWS, color features and automatic horizon, car, people, and face detectors. The labels for our training on this image are based on a threshold saliency map derived from human fixations (to the left of bottom right).

In contrast to manually designed measures of saliency, we follow a learning approach by using statistics and machine learning methods directly from eye-tracking data (Figure 2, simulation of human visual information processing mechanism). As shown in Figure 5, a set of low-level visual features are extracted from some training images. After the feature extraction process, the features of the top 5% (bottom 30%) points in the ground truth are selected as training samples in each training image. All of the training samples are sent to train a SVM model. Then, a test image can be decomposed into several feature maps and imported into SVM model to predict the saliency map. After the saliency map prediction, we can use them to obtain the human classification RoIs as inputs of CNN to continue solving classification problem.

After analyzing the EDOC dataset, we first extract a set of features for every pixel in each *m* × *n* pixels image. We recomputed the 35 features including 31 low-level features and 4 high-level features, for every pixel of the each image which resized to 200 × 200, and used these to train our visual attention model (Ours). The following are the low- level and high-level features (Figure 5) with which we were motivated to work after analyzing our dataset (Figure 2, simulation of human neural network).

*(1) Low-Level Features.* Because of the underlying biological plausibility [17], low-level features have been shown to correlate with visual attention. We use 31 low-level features:

- The local energy of the steerable pyramid filters [24] is used as features in four orientations and three scales (Figure 5, the first 13 images).
- We include intensity, orientation, and color contrast corresponding to image features as calculated by Itti and Koch's saliency [2] (Figure 5, images 14 to 16), because the three channels have long been seen as important features for bottom-up saliency.
- We include features used in a simple saliency model described by Torralba [25] and GBVS [21] and AWS [26] based on subband pyramids (Figure 5, images 17 to 19).
- The values of the red, green, and blue channels, as well as the probabilities of each of these channels, are used as features (Figure 5, images 20 to 25) in addition to the probability of each color as computed from 3D color histograms of the image filtered with a median filter at six different scales (Figure 5, images 26 to 30).
- The horizon is a place where humans naturally look for salient objects, because most objects rest on the surface of the earth. So we use the horizon as the last low-level feature (Figure 5, images 31).

*(2) High-Level Features.* In the light of the eye-tracking data obtained from our experiment, we found that humans fixated so consistently on people, faces, and cars, so we run the Viola Jones face detector [27] and the Felzenszwalb person and car detector [28] and include these as features to our model (Figure 5, images 32 to 35).

## 4. CNN for Feature Extraction

**Figure 6:**
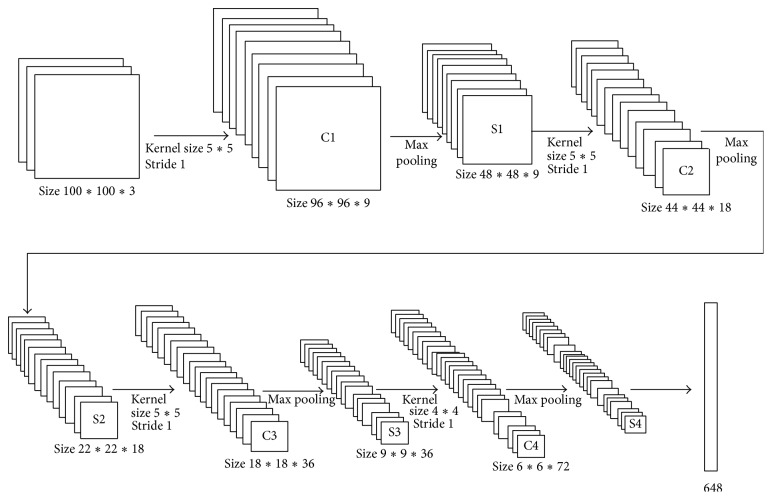
An illustration of the architecture of our CNN. The CNN we used contains 4 convolutional layers (C1~C4), the kernel sizes, respectively, are 5, 5, 5, and 4 pixels, the number of feature maps, respectively, is 9, 18, 36, and 72, and all of the stride is 1. All of the subsampling (S1~S2) size, respectively, is 2 pixels, and all of the stride is 1. The network's input is 3000 dimension features and output is 648 dimension features.

Convolutional neural network (CNN) was initially proposed by Cun et al. in the early 1980s [29]. Following the discovery of human visual mechanisms, local visual field is designed to make the CNN deep and robust in the 1990s. CNN is a neural network model, whose weight sharing network makes itself more similar to biological neural network, reducing the complexity of network model and the number of weight. CNN is based on four key architectural ideas: local receptive fields, convolution, weight sharing, and subsampling in the spatial domain. A CNN architecture is formed by a stack of distinct layers that transform the input volume into an output volume through a differentiable function. In a CNN structure, convolutional layers and subsampling layers are connected one by one and trained by supervised learning method with labeled data, the architecture of the CNN we used is shown in Figure 6, and the labeled data we used to train the CNN is obtained from our visual attention model. Due to the neuron network simulation, the CNN is usually used as a strong feature extractor and has achieved great success on image processing fields.

### 4.1. Convolution Layers

The convolutional layer is the core building block of a CNN. The layer's parameters consist of a set of kernels, which have a small receptive field, but extend through the full depth of the input volume. At a convolution layer, the previous layer's feature maps are convolved with learnable kernels and put through the activation function to form the output feature map. Each output map may combine convolutions with multiple input maps.

By training, kernels can extract several meaningful features; for example, the first convolutional layer is similar to Gabor filter, which can extract the information of corner, angle, and so forth. The CNN we used contains 4 convolutional layers (C1~C4), the kernel size, respectively, is 5, 5, 5, and 4 pixels, the number of feature maps, respectively, is 9, 18, 36, and 72, and all of the stride is 1 (Figure 6). Multilayers structure can abstract the input image layer by layer, to obtain a higher level distributed feature expression.

### 4.2. Subsampling Layers

Another important concept of CNNs is subsampling, which is a form of nonlinear downsampling. There are several nonlinear functions to implement subsampling among which max pooling is the most common. It partitions the input image into a set of nonoverlapping rectangles and, for each such subregion, outputs the maximum. The intuition is that once a feature has been found, its exact location is not as important as its rough location relative to other features.

A subsampling layer produces downsampled versions of the input maps. If there are* N* input maps, then there will be exactly* N* output maps, although the output maps will be smaller.

The CNN we used contains 4 subsampling layers (S1~S4), which are periodically inserted in between successive convolutional layers. All of the subsampling size, respectively, is 2 pixels, and all of the stride is 1 (Figure 6). Multilayers structure can abstract the input image layer by layer, to obtain a higher level distributed feature expression. By subsampling, we can not only reduce the dimension of features, but also improve their robustness.

### 4.3. Parameter Sharing

Parameter sharing scheme is used in convolutional layers to control the number of free parameters. It relies on one reasonable assumption; that is, if one patch feature is useful to compute at some spatial position, then it should also be useful to compute at a different position.

Since all neurons in a single depth slice are sharing the same parametrization, then the forward pass in convolutional layer can be computed as a convolution of the neuron's weights with the input volume. Therefore, it is common to refer to the sets of weights as a kernel, which is convolved with the input. Parameter sharing contributes to the translation invariance of the CNN architecture.

### 4.4. Fully Connected Layer

Finally, after several convolutional and max subsampling layers, the high-level reasoning in the neural network is done via fully connected layers and the CNN we used contains one fully connected layer. Neurons in a fully connected layer have full connections to all activations in the previous layer, as seen in regular neural networks. Their activations can hence be computed with matrix multiplication followed by a bias offset.

So far, the structure of our CNN network contains four convolutional layers, four subsampling layers, and one fully connected layer. We use the humans' classification RoIs obtained from visual attention model as the input of our CNN network, after feature extracting. Our CNN network outputs 648 dimension local features, which are parts of features used to classify objects.

## 5. Objects Classification

In order to be more close to humans' classification behavior, we build a task-based and learning-based visual attention model which combines low-level and high-level image features to obtain the humans' classification RoIs. Then, we construct CNN network to extract more features of those humans' classification RoIs. Although CNN is based on the neuron network simulation and is a strong feature extractor, the features obtained by CNN are the group of local features. However, humans always analyze images by putting them into context. Thus, for improving the biological advantages of our computer automatic classification method, we combine the 3 dimension high-level features also used in our visual attention model, including people, faces, and cars, with 648 dimension local features gained by our CNN network to classify objects.

Developing from statistics, the theory of SVM is a general learning method, which has excellent generalization ability in nonlinear classification, function approximation, and pattern recognition. Even though the sample is limited, SVM can effectively construct high-dimensional data model, can converge to the global optimum, and is insensitive to dimensions. Owing to the advantages of SVM, we use it to classify objects after acquiring 651 dimension features. The detailed processing of our classification method is shown in Figure 2.

## 6. Experimental Result and Discuss

In order to validate our classification method, we perform four experiments. (1) Section 6.1 evaluates our visual attention model (Ours) and compares it with other eight visual attention models. (2) Section 6.2 compares the classification results of using humans' classification RoIs as input of classification and using original images as input of classification. (3) Section 6.3 compares the classification results when only using features extracted by CNN and when combining high-level features and local features extracted by CNN. (4) Section 6.4 validates our classification method in 6000 images. In Sections 6.2, 6.3, and 6.4, we all use the error rate of classification and convergence rate as evaluation criterion. And our experiments were all based on a server IBM x3650m5, with CPU E5-2603v2 (2.4 GHz) and 32 GB RAM.

### 6.1. Performance of Our Visual Attention Model

**Figure 7:**
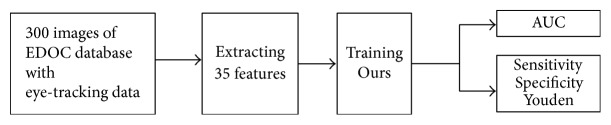
The whole processing of evaluating our visual attention model. We trained Ours after extracting features in EDOC database and measure Ours by AUC, sensitivity, specificity, and Youden and compared it with other eight visual attention models.

We validate our visual attention model by applying it to humans' classification RoIs prediction; the whole processing of this experiment is shown in Figure 7. We used EDOC database to evaluate our results; images were resized in 200 × 200 pixels. We randomly used 30 images of each class as training data and 20 images of each class as testing data. The database provided subjects' eye-tracking data as ground truth.

Since there is no consensus over a unique score for saliency model evaluation, a model that performs well should have good overall scores. We measure performance of saliency models in the following two ways.

First, we measure performance of each model by Area Under the ROC Curve (AUC). AUC is the most widely used metric for evaluating visual saliency. When AUC is equal to 1, the two distributions are exactly equal, not relative when AUC is equal to 0.5, and negatively relative when AUC is equal to 0.

Second, three quality measurements, classical sensitivity, specificity, and Youden, were computed. Sensitivity, also called the true positive rate, measures the proportion of positives which are correctly identified and is complementary to the false negative rate. The higher the sensitivity is, the more sensitive the test is. Specificity, also called the true negative rate, measures the proportion of negatives which are correctly identified and is complementary to the false positive rate. The higher the specificity is, the more precise the test is. Youden, called Youden, can be written as formula (1), whose value ranges from 0 to 1. The higher Youden is, the higher authenticity the test has. Besides, Youden gives equal weight to false positive and false negative values. Consider

(1) $$\begin{array}{c} \text{Youden}=\text{sensitivity}+\text{specificity}-1. \end{array}$$

#### 6.1.1. Analysis of AUC

Our method is biologically inspired. The developed method was compared with eight well-known techniques which dealt with similar challenges. These eight models were AIM [30], AWS [26], Judd [23], Itti [16], GBVS [21], SUN [19], STB [26], and Torralba [31]. We used them as the baseline because they also mimic the visual system. In the experiment, we randomly chose 30 images over the dataset of each class to train our model and the rest 20 images were used for testing. The statistical results are shown in Table 1.

**Figure 8:**
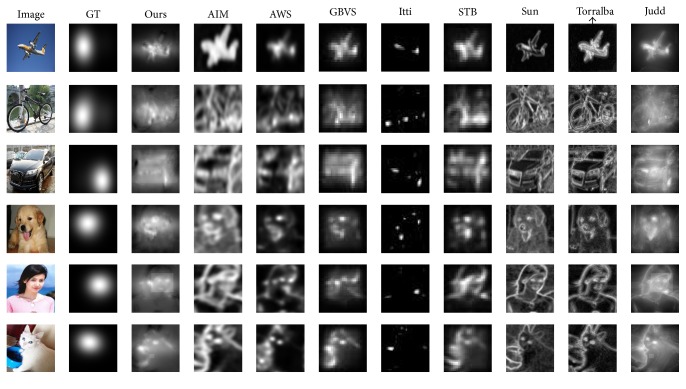
Some saliency maps are produced by 9 different models from the EDMERI database along with predictions of several models using ROC. Each example is shown by one row. From left to right: original image, ground truth, Ours, AIM, AWS, GBVS, Itti, STB, SUN, Torralba, and Judd. It is obvious that Ours is more similar to the ground truth than other saliency maps.

Table 1 shows the comparison of evaluation performances of the 9 models in the EDOC database. In this experiment, the average values of six classes in Table 1 are used for comparison. In the results, Ours has the best value in AUC. The AUC of our model is highest (0.8421), followed by Judd (0.8287) and GBVS (0.8284). However, the average is only 0.7642. It means the results of Ours are more identical with ground truth than other models. Generally speaking, Ours has good performance in this metric. And Figure 8 presents six examples of the saliency maps produced by our approach and the other eight saliency models.

#### 6.1.2. Analysis of Sensitivity, Specificity, and Youden

The ability of the different methods to predict humans' classification visual saliency maps was evaluated using conventional sensitivity, specificity, and Youden measurements. These results are shown in Table 2.

Table 2 shows sensitivity and specificity and Youden of the 9 models in 60% salient region. Overall, all sensitivity, specificity, and Youden measurements evidence that our model outperforms the other models. The sensitivity of our model is 73.2895%, which surpasses the average sensitivity 13.1638%, followed by Judd with 71.9905% and GBVS with 71.4794%. However, Itti had the lowest rate (only 40.8605%), less than approximately half of Ours. And the larger value of specificity (82.2354%) is also shown in our model, which exceeds the average specificity 4.0582%. Besides, the sensitivity of other models are all under 80% and under Ours. Owing to having the highest value of sensitivity and specificity, Youden (0.5552) of our model is the highest among the 9 models, followed by Judd with 0.967 and GBVS with 0.4934. Average Youden is 0.3830, which is only higher than half of Ours. The indisputable fact is that the higher Youden is, the higher authenticity the test has, and our model outperforms the other models in all sensitivity, specificity, and Youden measurements based on Table 2. Thus, Ours is suitable for predicting humans' classification visual saliency maps from images.

### 6.2. Comparison of Humans' Classification RoIs and Original Images

**Figure 9:**
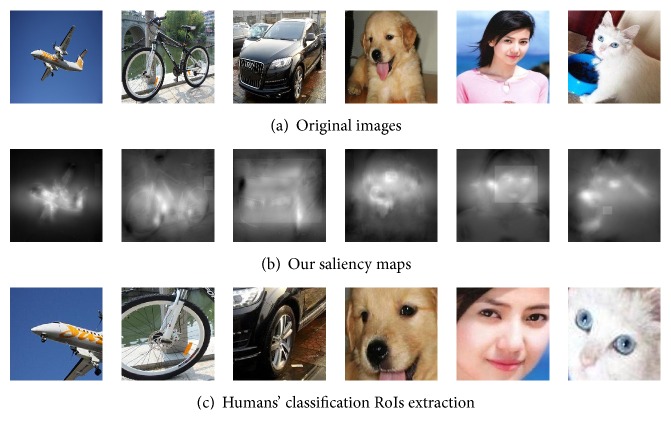
A sample of input of CNN. (a) is the original images of EDOC database. (b) is saliency maps acquired by our learning-based saliency model. We use (a) and (b) that can extract the humans' classification RoIs (c) which are the input of our CNN framework.

**Figure 10:**
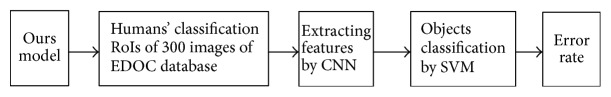
The whole processing of comparing the classification results when using humans' classification RoIs as input of classification and when using original images as input of classification. Different from traditional classification method using original images for classification, our classification method uses humans' classification RoIs as input of classification. After extracting features by CNN, we use SVM model to classify objects and then use the error rate of classification and convergence rate as evaluation criterion.

To testify that humans' classification RoIs outperform original images in objects classification, we used humans' classification RoIs (Figure 9) obtained by the original images of EDOC database and humans' classification visual saliency maps to classify objects and then compare the result of classification with outcome of the experiment when using the original images of EDOC database as input of classification. All images were resized in 100 × 100 pixels. We input two groups of images, the original images and humans' classification RoIs to our CNN framework to extract features. As introduced above, the architecture of the CNN we used contained 4 convolutional layers and 4 subsampling layers. For two groups of images, we input them to CNN by 3 times, and the frequency of training of CNN is, respectively, 500, 1000, and 1500. We randomly used 30 images of each class as training data and 20 images of each class as testing data. Finally, we used SVM to classify objects and used the error rate to check out whether using humans' classification RoIs can make the classification results better and the whole processing of this experiment as Figure 10 showed. The error rates of the classification results by three different frequency trainings, 500, 1000, and 1500 in two groups of input images, including original images and humans' classification RoI, are shown in Table 3.

Table 3 demonstrates the error rate of the experiments' result by three different frequency trainings in two groups of input images. Overall, all results evidence that our method based on humans' classification RoIs exceeds traditional CNN based on original images. Although when the frequency is 500, the error rate of two method is all more than 50%, our method is less than traditional method 10%. With the increasing of the frequency of training, the error rate of our method based on humans' classification RoIs drops quickly from 63.3% to 18.2%. However, the error rate of traditional CNN based on original images is 50% when the frequency of training is 1000, and even when the frequency of training is 1500, it is still more than 30%. Besides, along with increasing of frequency of training, the error rate will be lower. As we all know, the error rate is lower, and the results of classification are better. So it is not denied that humans' classification RoIs can make the results of classification better.

### 6.3. Combining High-Level Features and Features Extracted CNN

**Figure 11:**
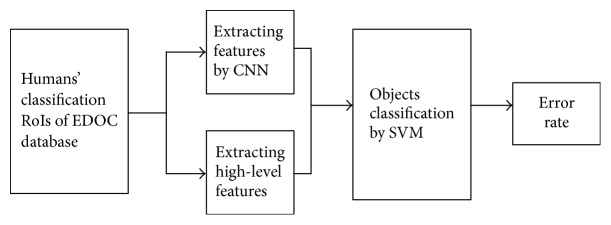
The whole processing of combining high-level features and features obtained by CNN to classify objects. Before SVM model, we added high-level features and then use the error rate of classification and convergence rate as evaluation criterion.

To prove that combining high-level features and local features extracted by CNN can make the results of classification better, we performed an experiment which added high-level features to SVM model to classify objects and then compared the classification's result of experiment 6.2. And the other settings of experiment 4 were the same as experiment 3 and the whole processing of this experiment as Figure 11 showed. And the comparison of error rate of the classification's results in two features extracting ways is shown in Table 4.

Table 4 shows the comparison of error rate of the classification's results in two features extracting ways. On balance, all results evidence that the classification way based on combining two types of features exceeds only based on features extracting by CNN. When the frequency is 500, the error rate of comprehensive method is 51.7%, which is less than the single method nearly 12%. According to Table 4, we can conclude that the bigger frequency of training is, the lower error rate will be. But when the frequency of training is 1000, the error rate of comprehensive method (25.8%) is also less than the single method (36.7%) nearly 10%. Most of all, when the frequency of training is 1500, the error rate of comprehensive method (14.2%), which is less than the single method 4%, is almost half of the error rate of the method (36.5%) using original images according to the Table 3. Hence, adding high-level features can make the classification results better.

### 6.4. Performance of Our Classification Method in 6000 Images Classification

**Figure 12:**
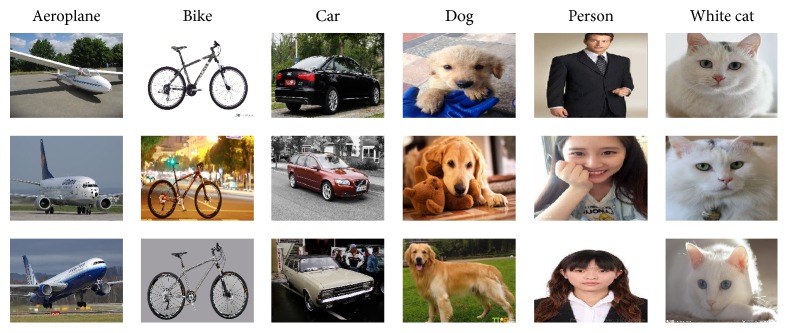
Images. A sample of the 6000 images of ImageSix database.

**Figure 13:**
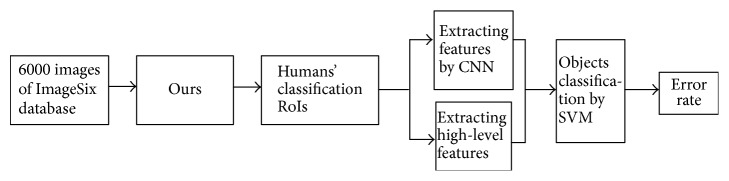
The whole processing of our classification method validated by ImageSix database. First, we predicted humans' classification RoIs of original images in ImageSix database. Second, we combined the features extracted by CNN and high-level features to classify objects by SVM and then used the error rate of classification as evaluation criterion.

Sections 6.1, 6.2, and 6.3 are all based on the 300 images of EDOC database; the numbers of images are not big, but there is not suitable and available big database including the six classes objects to testify the robustness of our classification method. Thus, we construct big database ImageSix (Figure 12), including 6000 images from the Internet. Firstly, we used Ours to predict the humans' classification RoIs of the images of ImageSix database. Secondly, we extracted the local features of these humans' classification RoIs by CNN. Then, we combined these local features with high-level features extracted by Ours to perform three classification experiments by SVM, and the frequency of training of CNN was also, respectively, 500, 1000, and 1500. For SVM, we randomly used 600 images of each class as training data and 400 images of each class as testing data. The whole processing of this experiment is shown in Figure 13. Finally, we compared the classification's results of our method with outcome of classification method which used the original images as input and extracted features only based on CNN, and the experiment's results are shown in Table 5.

Table 5 shows the error rate of the experiments' result by two methods in ImageSix database. From it, we can conclude that, with the increasing of the number of the training images, the error rate of two methods both drop, but all results evidence that our classification method exceeds the classification method without improvement. When the frequency is 500, the error rate of our method is 44.6%, which is less than classification method without improvement (63.2%) nearly 20% and is even less than the error rate of classification method without improvement (56.7%) in the 1000 frequencies of training. With increasing of frequency of training, the error rate will be lower. However, when the frequency of training is 1500, the error rate of classification method without improvement is still more than 47%. With the increasing of the frequency of training, the error rate of our method drops quickly from 44.6% to 29.1%. Thus, our classification method can make the classification results better without doubt.

## 7. Conclusion and Discussion

The present paper has introduced a new classification method which combines learning-based visual saliency model and CNN. This method inspired the completed processing that humans classify different kinds of objects and has apparently advantages in biology.

Firstly, we established a database, called EDOC, to learn common people visual behaviors and record their eye-tracking data when they classify different objects.

Secondly, we built a learning-based visual saliency model trained by EDOC database. Our model has the ability to automatically learn the relationship between saliency and features. And our model simultaneously considers appearing frequency of features and the pixel location of features, which intuitively have a strong influence on saliency. As a result, our model can determine saliency regions and predict humans' classification RoIs more precisely.

Then, we built a CNN framework and used humans' classification RoIs obtained from our visual attention model to train CNN; thus, it will be closer to humans.

Finally, for improving the biological advantages of our computer automatic classification method, we combined the 3 dimension high-level features with 648 dimension local features gained by our CNN network to classify objects by SVM.

To evaluate every aspect of our classification method, we performed 4 groups of experiments. In particular, we established a big ImageSix database, including 6000 images to testify the robustness of our classification method. And all experimental results showed that our method made the efficiency of classification improve significantly.

Our classification method is inspired by the completed processing that humans classify different kinds of objects; however, it is not denied that human thinking process is so sophisticated; we cannot copy the full processing. Besides, for different objects, human thinking process is quite different. So, in the future, to improve the performance of our method, we can optimize the processing of feature extraction and build different CNN framework for different objects; meanwhile, it will become very costly.

## Figures and Tables

**Table 1:** Performance comparison of nine models in the EDOC dataset.

Metrics	GT	Ours	AIM	AWS	GBVS	ITTI	STB	SUN	Torralba	Judd	Average
AUC	1.0000	0.8421	0.7232	0.7811	0.8284	0.6078	0.8151	0.7360	0.7158	0.8287	0.7642

**Table 2:** Sensitivity, Specificity, and Youden of nine models.

Metrics	Ours	AIM	AWS	GBVS	ITTI	STB	SUN	Torralba	Judd	Average
Sensitivity (%)	73.2895	51.8212	61.1878	71.4794	40.8605	67.9153	49.8252	52.7619	71.9905	60.1257
Specificity (%)	82.2354	77.4174	79.1480	77.8588	77.8217	78.8674	76.0314	76.5402	77.6745	78.1772
Youden	0.5552	0.2923	0.4034	0.4934	0.1868	0.4678	0.2586	0.2930	0.4967	0.3830

**Table 3:** The error rate of the classification results by three different frequency trainings of CNN in two groups of input images.

Input images	Frequency of training	500	1000	1500
300 original images	Error rate%	73.3	50.0	36.5
Humans' classification RoIs		63.3	36.7	18.2

**Table 4:** The comparison of error rate of the classification's results in two features extracting ways.

Features	Frequency of training	500	1000	1500
Features extracting by CNN	Error rate%	63.3	36.7	18.2
High-level features and features extracting by CNN		51.7	25.8	14.2

**Table 5:** The error rate of the classification results by three different frequency trainings of CNN in two groups of input images.

Method	Frequency of training	500	1000	1500
Without improvement	Error rate%	63.2	56.7	46.9
Our classification method		44.6	33.8	29.1
